# Can Healthcare Assistant Training (CHAT) improve the relational care of older people? Study protocol for a pilot cluster randomised controlled trial

**DOI:** 10.1186/s13063-015-1077-3

**Published:** 2015-12-09

**Authors:** Antony Arthur, Jill Maben, Heather Wharrad, Clare Aldus, Sophie Sarre, Justine Schneider, Caroline Nicholson, Garry Barton, Karen Cox, Allan Clark

**Affiliations:** School of Health Sciences, Edith Cavell Building, Faculty of Medicine and Health Sciences, University of East Anglia, Norwich, NR4 7TJ UK; Florence Nightingale Faculty of Nursing and Midwifery, King’s College London, James Clerk Maxwell Building, 57 Waterloo Road, London, SE1 8WA UK; School of Health Sciences, Queen’s Medical Centre, Faculty of Medicine and Health Sciences, University of Nottingham, Nottingham, NG7 2HA UK; School of Sociology and Social Policy, Law and Social Sciences Building, University of Nottingham, Nottingham, NG7 2RD UK; Norwich Medical School, Faculty of Medicine and Health Sciences, University of East Anglia, Norwich, NR4 7TJ UK; Norwich Clinical Trials Unit, Faculty of Medicine and Health Sciences, University of East Anglia, Norwich, NR4 7TJ UK

**Keywords:** Pilot, Feasibility, Cluster randomised controlled trial, Older people, Healthcare assistants, Nursing, Hospital care, Empathy, Training

## Abstract

**Background:**

People aged 75 years and over account for 1 in 4 of all hospital admissions. There has been increasing recognition of problems in the care of older people, particularly in hospitals. Evidence suggests that older people judge the care they receive in terms of kindness, empathy, compassion, respectful communication and being seen as a person not just a patient. These are aspects of care to which we refer when we use the term ‘relational care’. Healthcare assistants deliver an increasing proportion of direct care to older people, yet their training needs are often overlooked.

**Methods/Design:**

This study will determine the acceptability and feasibility of a cluster randomised controlled trial of ‘Older People’s Shoes’ a 2-day training intervention for healthcare assistants caring for older people in hospital. Within this pilot, 2-arm, parallel, cluster randomised controlled trial, healthcare assistants within acute hospital wards are randomised to either the 2-day training intervention or training as usual. Registered nurses deliver ‘Older People’s Shoes’ over 2 days, approximately 1 week apart. It contains three components: experiential learning about ageing, exploration of older people’s stories, and customer care. Outcomes will be measured at the level of patient (experience of emotional care and quality of life during their hospital stay), healthcare assistant (empathy and attitudes towards older people), and ward (quality of staff/patient interaction). Semi-structured interviews of a purposive sample of healthcare assistants receiving the intervention, and all trainers delivering the intervention, will be undertaken to gain insights into the experiences of both the intervention and the trial, and its perceived impact on practice.

**Discussion:**

Few training interventions for care staff have been rigorously tested using randomised designs. This study will establish the viability of a definitive cluster randomised controlled trial of a new training intervention to improve the relational care proided by healthcare assistants working with older people in hospital.

**Trial registration:**

The study was registered as an International Standard Randomised Controlled Trial (ISRCTN10385799) on 29 December 2014.

## Background

Older people account for a large and increasing proportion of those receiving National Health Service (NHS) acute care. In England in 2013, people over the age of 75 years accounted for 24 % of all hospital admissions, an increase of 57 % over the previous decade with the average hospital stay for this age group decreasing from 15.2 to 9.4 days [1]. The quality of care delivered to older people has come under increased scrutiny: a report by The King’s Fund cites 32 initiatives from statutory bodies, charities and campaign groups drawing attention to deficiencies in their care [2]. The King’s Fund’s Point of Care Programme was a response to a more general concern about ‘not getting the basics right’ in the delivery of care for older people [3, 4].

Just under a fifth of respondents to the NHS Inpatient Survey did not feel that they were treated with respect and dignity at all times [5] and in complaints received about NHS care, the second highest area of concern related to the attitudes of staff [6]. Recently, the Care Quality Commission (CQC) review of services in 2012 found that they were ‘struggling in areas such as dignity and respect, nutrition, care and welfare’ [7] and the Patients Association published 13 cases of care failures [8]. The Prime Minister has acknowledged this situation by prioritising the improvement of care standards in 2013 [9].

While patient-centred care is an explicit priority, there is a lack of clarity among staff at all levels as to what this actually means and how it can be practically implemented [10]. Emotional support, empathy and respect are the aspects of care considered most important by patients [11]. Key elements of dignified care include respectful communication, respecting privacy, promoting autonomy, addressing basic needs in a sensitive manner, and promoting a sense of identity [4]. Qualitative data from a study of older patients with acute care needs has highlighted the importance of timeliness of care (particularly around toileting needs) and interest in the person, kindness, compassion and attending to ‘the little things’ [12].

The focus of the proposed study is the relational care provided to older people in hospital. Relational aspects of care include dignity, empathy and emotional support as distinct from functional or transactional aspects of care [13]. In a review of studies of older people and their relatives’ experiences of acute care settings, it was the relational aspects of care that affected whether care experiences were perceived as good or bad [14]. Three themes that underscored older people’s understanding of relational care were identified in this review: older people’s need for reciprocity (‘connect with me’); maintaining their identity (‘see who I am’); and sharing decision-making (‘include me’). There is now a substantial body of evidence from which to conclude that older people place great importance on the relational aspects of their care and, when this falls short, its absence is felt most acutely.

Perhaps due to the nature of the work they do, nurses have often been targeted as both the source of the problem and the solution to concerns about loss of dignity for patients in hospital [3]. However, within the NHS, Band 2 and Band 3 support workers, also known as healthcare assistants (HCAs), have become an increasingly important section of the workforce, particularly in relation to older people, with observational data suggesting that the proportion of their time delivering direct and indirect patient care is approximately 60 %, nearly twice that of registered nurses [15]. Demographically, HCAs tend to differ from registered nurses, more closely resembling the ethnic diversity of the patient population they serve [16] and likely to be a more ‘static’ part of the workforce. The problems of invisibility, marginalisation and subordination of the ‘caring’ work of nurses [17] are likely to be replicated in HCAs whose work often gets little recognition, even from other staff groups [18].

Although investments in staffing and work environments are pre-requisites for high-quality care [12, 19], historically HCAs have been viewed as the ‘untrained workforce’ leading to an assumption that they are without training needs [20]. HCAs and nurses are largely in favour of more formal training for HCAs, although a blurring of role boundaries is of concern to both staff groups [21]. Among employing organisations there is a lack of consistency in HCA training and how HCAs interface with registered nurses [22]. Moreover, it appears that HCAs often lack confidence in pursuing the few training opportunities available to them [16, 18]. Ethnographic observational data of HCAs working in dementia wards suggest that support in carrying out such a challenging role is drawn from the formation of close-knit groups of HCAs who are sometimes disconnected from the wider ward team [23], resulting in HCAs feeling alienated from the organisation in which they work [24].

Training of HCAs has hitherto been ad hoc, variable, and marked by a tendency to focus on tasks and competencies, with little attention paid to relational care. The importance of using principles of instructional (pedagogical) design [25] to develop educational and training interventions is rarely considered. This is essential to ensure that training builds on existing knowledge and values, harnesses intrinsic motivation, and actively engages learners.

To date, evaluations of training interventions for HCAs have been few, are typically lacking any comparative element (e.g. [26]), and are often small-scale. In terms of Kirkpatrick’s four-level training evaluation model [27], studies of HCA training rarely measure outcomes other than those that are first-level and ‘reactive’ (for example trainee satisfaction) and greater efforts should be made to see how training translates into fourth-level benefits or ‘results’ (for example patient outcomes). This study will pilot ‘Older People’s Shoes’, a newly developed evidence-based HCA training package designed to improve the quality of relational care of older people and investigate the feasibility of testing its effectiveness in a definitive randomised controlled trial.

### Aims of the study

The aims of the study are to assess the feasibility of a cluster randomised controlled trial to compare the performance of an HCA training package in relational care against current training in improving the care of older patients in acute NHS settings, and to explore optimal methods for cost-benefit analysis for a definitive study.

Important parameters that are needed to inform the feasibility of a definitive trial (and if feasible, then the design of such a trial) [28] will be assessed:

1. The acceptability of the intervention to trainers and HCA trainees.
2. The willingness of ward managers, HCAs and older patients to participate in a cluster randomised controlled trial.
3. The willingness of ward managers for wards to be randomly allocated.
4. The level of non-response and item non-response to outcomes at the level of ward, HCA and patient.
5. The acceptability of outcome measures to participants.
6. The ability to monitor levels of resource-use and quality of life data.
7. The variability within and between ward, HCA and patient outcomes.
8. The appropriateness of ward as the unit of randomisation.

## Methods/Design

### Trial design

**Fig. 1:**
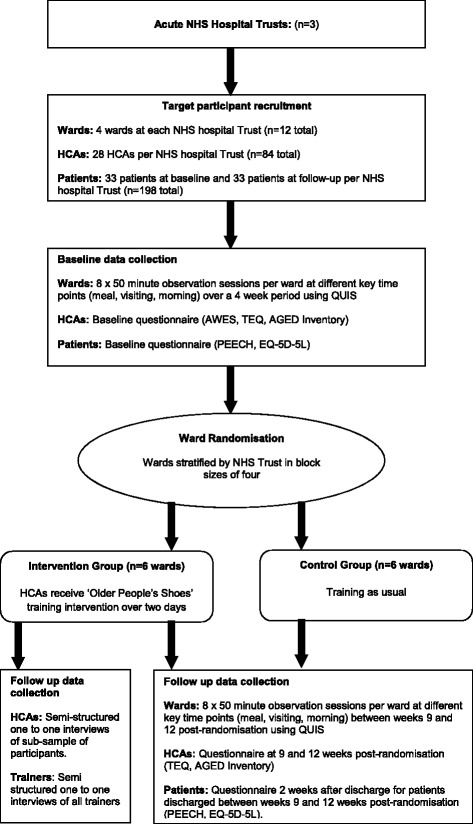
Can Healthcare Assistants Training improve the relational care of older people? (CHAT) pilot cluster randomised controlled trial design

A pilot cluster randomised controlled trial will be conducted to compare an ‘HCA training package in relational care’ with ‘HCA training as usual’. Clusters are wards within three acute NHS Hospital Trusts in England with outcomes observed at the level of ward, HCA and patient (Fig. 1).

### Eligibility criteria

#### Wards

General medical, stroke or wards for the care of older people are eligible to enter the trial. Specialist dementia wards and medical admissions units are excluded.

#### Healthcare assistants

HCAs employed full time or part time within enrolled wards are eligible to enter the trial. Those employed as bank staff and not part of the named staff on the ward roster are ineligible.

#### Patients

Patients will be eligible if they are aged 70 years or over and discharged from an inpatient stay on an enrolled ward, during either the 4-week period prior to randomisation (baseline) or during weeks 9 to 12 post-randomisation (follow-up). Patients transferred to another ward or hospital prior to discharge or considered by the nurse-in-charge not to have mental capacity (according to the Mental Capacity Act 2005) or to be in the final stages of a terminal illness are excluded.

### Recruitment

#### Wards

The ward manager provides permission for ward participation. Recruitment of wards will cease once permission is given by ward managers of 4 eligible wards from each of 3 acute NHS Hospital Trusts (*n* = 12 wards in total).

#### Healthcare assistants

Within each of the enrolled wards all HCAs will be invited to take part in the study by a researcher employed on the grant. At a number of ward-based meetings during the 4-week baseline period HCAs are given information about the study. Informed consent will be obtained from all HCA participants.

#### Patients

The initial approach of patients will be made on the enrolled ward a few days prior to their discharge. Older patients (aged 70 years or over) receiving inpatient care from the enrolled wards in the 4-week baseline period and the 4-week follow-up period will be identified by a hospital-based research nurse in consultation with ward managers. Informed consent will be obtained from all patient participants. The research nurse will approach each of the patients meeting eligibility criteria, explain the study, and provide the patient with a participant information sheet. If they agree to receive a questionnaire after discharge from hospital, the research nurse will ask them to sign a consent form.

### Baseline measures

#### Wards

To assess quality of interactions within a ward the Quality of Interaction Schedule (QUIS) observation tool is used by a trained observer at each hospital [29]. QUIS is an observational strategy in which interactions between patients and care staff are coded as positive social (interactions involving conversation and companionship), positive care (interactions during the appropriate delivery of care), neutral (brief and indifferent interactions), negative protective (keeping safe without explanation or reassurance) or negative restrictive (opposing or resisting patients’ freedom of action without good reason). Ward observations take place over the 4-week period prior to randomisation. Each observation is conducted over a 50-minute period by 1 observer. The interactions observed are those that involve a patient and at least one HCA. Eight observations per ward will be conducted during the baseline period. Observations take place during mornings, mealtimes and visiting periods. On four occasions at each hospital, observations will be conducted in pairs to assess inter-rater reliability.

#### Healthcare assistants

At baseline, HCAs receive a self-completion questionnaire containing the Assessment of Work Environment Schedule (AWES), [30, 31] the Toronto Empathy Questionnaire (TEQ) [32], and the Age Group Evaluation and Description (AGED) inventory [33]. The 34-item AWES measures HCA perception of the support provided in the work environment. The TEQ conceptualises empathy as an emotional process and contains 16-items, each a statement about empathetic responses to specific situations with which the HCA respondent is asked to rate on a 5-point scale their agreement. The AGED inventory measures the extent to which stereotypes about ageing are held by the respondent. It includes 28 semantic differentials relating to a specific age group (70 years and over) using a 7-point Likert scale.

#### Patients

At 2 weeks after discharge from hospital, patients who have consented to participate receive a questionnaire and pre-paid addressed envelope. To assess the relational aspects of care experienced by patients, the Patient Evaluation of Emotional Care during Hospitalisation (PEECH) [34, 35] is used. The PEECH was developed for acute hospital settings and contains 23 items and 4 subscales of levels of security, knowing, personal value and connection. Patients are asked to rate the extent (on a four-point scale) to which hospital staff responded or behaved in particular situations. To assess quality of life, the self-report version of the European Quality of Life 5 Dimensions 5 Levels (EQ-5D-5L) [36] is used.

### Allocation for trial interventions

Stratified by NHS Hospital Trust, 12 wards will be randomly allocated by the Norwich Clinical Trials Unit. Each ward has an equal chance of receiving either training for HCAs in relational care or training as usual. Random allocation is generated via computer-written code. To conceal allocation from those responsible for recruitment, randomisation takes place immediately after baseline measures are completed and 4 weeks ahead of the start of the intervention (set-up period) to allow appropriate arrangements including HCA cover.

### Intervention

#### HCA training in relational care: Older People’s Shoes

HCAs from wards randomised to the new training intervention (*n* = 6 wards, 2 wards per hospital) will receive ‘Older People’s Shoes’, a newly developed HCA training package that focuses on the relational care of older people. The training intervention was developed using evidence from interviews with HCAs, other ward staff, older people, and a panel of experts. The intervention development work was funded by the same grant but in an earlier phase of this project. Course materials comprise a trainer manual and trainee workbook, PowerPoint resources and an online version of the training.

Training will take place during weeks 5 to 8 post-randomisation after the 4-week set-up period. It comprises 2 training days approximately 1 week apart. Older People’s Shoes training is delivered by registered nurses, all of whom are employed at the local Hospital Trust in practice development or education and training roles. These trainers receive full training in the content and delivery of ‘Older People’s Shoes’ from members of the research team.

Training comprises three units relating to the care of older people in hospital:

1. Walking in Older People’s Shoes.
  This unit helps HCA learners to understand the challenges of being an older person in hospital. Patients’ experiences are brought to life using videos and narratives in which real older patients talk about their experiences (both good and bad) of hospital care. Experiential learning is provided through learners having the opportunity to use age simulation suits.
2. Getting to know older people.
  This unit challenges HCA participants to think about how hospitalisation can strip away much of what makes people individual; and how stereotypical notions of ageing may lead care staff to make false or limiting assumptions about older people. It looks at how opportunities can be found to ‘discover the person behind the patient’ through life stories, and so build stronger relationships with the older people they care for.
3. Learning from customer care.
  This unit asks HCA participants to consider how some aspects of customer care provided in non-health settings can be applied to their work in the ward. This includes ‘active listening’ and reading body language to try to understand what may lie behind the behaviour of patients that are seen as ‘difficult’; and how to deal with angry patients or visitors. The aim is that learners will come to understand how relatively simple techniques can be incorporated into their own practice. The unit also addresses the need for HCAs to look after themselves.

Each unit is divided into two sessions, one per day, so that learning on the first day can be consolidated and developed during the second day. At the end of Day 1 HCAs are asked to undertake brief individual work-based exercises prior to Day 2. Additional materials are also available online with access restricted to HCAs allocated to the training intervention.

Training seeks to promote empathy with older patients, give time for reflection and shared experience, affirm the importance of the HCA role, and ensure that HCAs know how to access their local support network for any issues that may arise as a result of the training intervention. Given the practical, hands-on nature of much of an HCA’s work, the overarching theoretical basis for the training package is derived from Carver’s framework for understanding experiential education [37]. Carver’s four key elements to experiential education are applied throughout the training intervention:

1. Authenticity – activities are directly relevant to the participant HCA’s role in caring for older people in hospital.
2. Active learning – group exercises are embedded throughout to maintain HCA participant engagement and ensure learning is active rather than passive.
3. Drawing on experience – HCA participants are encouraged to think about what happened to them in particular situations, how it felt, how they reacted, what resulted, and what they observed.
4. Provision of mechanisms for connecting experience to future opportunity – HCA participants are encouraged to reflect on their participation in learning activities to make their experiences relevant to their future work with older people.

The pedagogical approach used to inform the design of the training intervention was Gagne’s nine-step model [25] (gain attention, identify objective, recall prior learning, present stimulus, guide learning, elicit performance, provide feedback, assess performance, enhance retention/transfer). The ' Older People’s Shoes' training intervention used this model in a reduced form to structure individual learning activities: learning objective, trigger, content, guided practice/reflection, key messages.

### Training as usual

HCAs from wards not randomised to the training intervention (*n* = 6 wards, 2 wards per Trust) will receive ‘training as usual’. This is typically restricted to periods of staff induction or focussed on mandatory training requirements such as manual handling. HCAs will receive no training in relational care beyond any that might be experienced as part of the standard process within their employing NHS Hospital Trust. Educational leads for each NHS Hospital Trust have provided details of how this is operationalised for HCAs within their organisation.

### Outcomes

Outcomes at all levels (ward, HCA and patient) will be observed during weeks 9 to 12 after randomisation. The primary outcome is at the level of patient (PEECH score).

#### Wards

Between weeks 9 and 12 post-randomisation, 8 observations per ward will be conducted using identical methods to those used in the baseline period.

#### Healthcare assistants

HCAs will receive the TEQ and AGED inventory twice during the follow-up period, at weeks 9 and 12 post-randomisation.

#### Patients

Patients due to be discharged from enrolled wards between weeks 9 and 12 post-randomisation will be approached, recruited and administered questionnaires in an identical way to that used in the baseline period.

#### Measures of cost and cost-effectiveness

HCA staff in the intervention arm will be asked in the follow-up questionnaire whether the average duration of their patient contact times has changed since they received the training in relational care. Ward records will be used to ascertain the number of days patients stayed in the ward. Associated analysis will focus on completion rates in order to identify the feasibility of collecting such data, and inform the design of any future definitive study. Appropriate unit costs (e.g. Curtis [38]) will also be attached to all items of resource-use in order to enable the overall costs to be estimated and thereby identify important cost drivers. Completion rates for the EQ-5D-5 L will be used to assess whether it is appropriate for this population group, and the extent to which a future definitive cluster randomised controlled trial would be better designed as a cost-consequences analysis, where the incremental cost would be presented in relation to ward, HCA and patient outcomes as appropriate.

#### Qualitative interviews of trainers and HCAs

Following the delivery of the intervention, trainers will be asked to undertake a semi-structured qualitative interview. These interviews will follow a topic guide covering specific aspects of delivering the intervention and more general aspects about learner engagement. Directly after the follow-up period a sub-sample of HCAs who have undertaken the training intervention will be asked to undertake a semi-structured qualitative interview. These interviews will follow a topic guide covering experiences of both the training intervention itself and being part of the pilot trial.

All qualitative interviews are expected to last between 30 and 45 minutes. They will be conducted by a member of research staff, and be audio-recorded if given permission to do so by the interviewee.

### Sample size

As the study is to test feasibility and is a pilot cluster randomised controlled trial, it is not powered to determine superiority of HCA training in relational care or training as usual.

#### Wards

Observations by a researcher employed on the grant will take place on the four enrolled wards at each participating NHS Hospital Trust. For each ward, eight observations will take place during the baseline period and eight during the follow-up period. Each observation will last for 50 minutes.

#### HCAs

All eligible HCAs will be invited to take part. Numbers of HCAs employed on wards varies within and between NHS Hospital Trusts. Assuming approximately 10 HCAs employed on each enrolled ward, and an estimated recruitment rate of 70 %, it is anticipated that 84 HCAs will be recruited (42 per arm). A total of 12 HCAs from the intervention arm will be invited to take part in a semistructured interview. Anticipating that there will be some shortfall between recruitment to training and uptake of training, this number represents approximately one third of expected trainees. Requesting interviews with a greater number would put undue strain on ward staffing levels. Of those HCA trainees who give initial consent to interview, purposive sampling will be used to select across NHS Hospital Trusts, and to maximise variation of interviewees in terms of gender and length of experience.

#### Patients

It is anticipated that across all 3 NHS Hospital Trusts 100 patients will receive questionnaires during the 4-week baseline period and a further 100 patients will receive questionnaires during the 4-week follow-up period.

#### Trainers

All trainers (one or two per NHS Hospital Trust) who deliver the training intervention will be asked to take part in follow-up semi-structured interviews.

### Fidelity

All training intervention sessions will be observed by at least one member of the research team. Deviations from the trainer guide will be recorded. In addition, one member of the research team will observe the training intervention being delivered at each of the three centres to record differences in delivery between trainers. For those HCAs who attend the training intervention days, the number of days actually attended will be recorded.

### Statistical analysis

Within the analysis plan, the emphasis will be on the estimation of parameters required for a future sample size calculation and potential differences via confidence intervals, rather than formal hypothesis testing.

#### Ward-level analysis

QUIS will be analysed as a total mean rating for each observed session. Analysis will be based on the change from baseline to outcome using a general linear model including NHS Hospital Trust as this was a stratification variable. Due to the small number of wards this analysis will be descriptive.

#### HCA-level analysis

The outcomes of TEQ and AGED will be assessed using a linear mixed-effect model, with fixed effect being the intervention and the random effect will be ward. This will account for the potential of dependence of HCA-level responses from HCAs within the same ward. Additionally, the baseline value of the outcome will also be included as a fixed effect in a sensitivity analysis. These models will allow the estimation of the parameters required, including the within-ward and between-ward variance, for the planning of future trials, including the HCA-level variation and between-ward variation.

Due to the small number of clusters, the results of the random-effect model will also be compared to those using a generalised estimating equation (GEE) model. Additionally, as the number of clusters is less than 15 per arm [39], sensitivity will be analysed as a total average, per ward, rating as well as the individual sub-types. Due to the small number of wards involved this analysis will be descriptive. Additionally, the sensitivity of the assumption of a normally distributed outcome will be assessed using the two-stage, non-parametric bootstrap.

#### Patient-level analysis

All analysis will be based on the intention-to-treat principle, including all recruited patients from within randomised wards. The total PEECH score will be analysed using a linear mixed-effect model with fixed effect being the intervention and the random effect will be ward in order to account for the potential of dependence of patient-level responses from patients within the same ward. The four subscales will be analysed using the same model. These models will allow the estimation of the parameters required for the planning of future trials, including the patient-level variation and between-ward variation. As with the HCA-level analysis, for the patient-level outcomes we will compare results from the random-effect model with those from a GEE model. The sensitivity of the assumption of a normally distributed outcome will be assessed using the two-stage non-parametric bootstrap.

If appropriate, sensitivity of the results to missing data will be checked via multiple imputation. If appropriate, adjustment for baseline factors will be made.

### Qualitative analysis

The qualitative interview data will be analysed thematically. As the purpose of these interviews is to inform the understanding of the process of both the trial and the delivery and receipt of the intervention, we will use framework analysis [40]. This is a method that is particularly useful for applied research designed to meet specific information needs yet remains true to the accounts of the interviewees.

### Trial Steering Committee

A Trial Steering Committee (TSC) will monitor and supervise the pilot cluster randomised controlled trial in accordance with National Institute of Health Research Health Services and Delivery Research (NIHR HS&DR) Research Governance Guidelines for Trial Steering Committees (May 2013). The TSC comprises: Professor Karen Spilsbury, (TSC independent chair); Professors Antony Arthur, Jill Maben and Heather Wharrad (study co-applicants); Professor Jackie Bridges, Dr Tanis Hand, Dr Gail Adams, Dr Bev Fitzsimons and Dr Lynne Williams (expert advisors), Mrs Margaret McWilliams, Mrs Janet Gray and Ms Sagila Thiruthanikasalan (advisors representing the views of health service users and HCAs); Dr Ines Mesa Eguiagaray (independent statistician), Dr Ella Zomer (independent health economist).

### Ethical approval

A favourable ethical opinion for this study (CHAT (feasibility randomised controlled trial) Protocol v2 9.2.2015) was granted by Cambridge South Research Ethics Committee on 13 February 2015 (application number 15/EE/0025, CSP reference162616). Full NHS Research and Development approvals were granted for each study site (King’s College Hospital NHS Foundation Trust 17 March 2015; Norfolk and Norwich University Hospitals NHS Foundation Trust, 19 March 2015; Nottingham University Hospitals NHS Trust, 15 April 2015). The study has been adopted onto the UK CRN portfolio (study ID UKCRN18280).

## Discussion

We have made the following modification to the trial. Our ward observational tool is now the QUIS rather than the Care Kindness and Compassion Observation Tool [41] as originally specified (ISRCTN10385799). Wider use of the QUIS in research settings including another existing NIHR funded study of compassionate care in hospital wards [42] informed this decision. This change has been agreed by the study sponsor, funder and has received ethics committee approval.

## Trial status

To date, 12 wards have been enrolled into the study, 112 HCAs have consented to take part and 115 patients have consented to receive questionnaires.

## Abbreviations

AGED: Age Group Evaluation and Description
AWES: Assessment of Work Environment Schedule
CHAT: Can Healthcare Assistants Training improve the relational care of older people?
CQC: Care Quality Commission
EQ-5D-5L: European Quality of Life 5 Dimensions 5 Levels
GEE: generalised estimating equation
HCAs: healthcare assistants
NHS: National Health Service
NIHR HS&DR: National Institute of Health Research Service and Delivery Research
PEECH: Patient Evolution of Emotional Care during Hospitalisation
QUIS: Quality of Interaction Schedule
TEQ: Toronto Empathy Questionnaire
TSC: Trial Steering Committee
